# Statistical design of mental health complex intervention trials

**DOI:** 10.1186/1745-6215-12-S1-A145

**Published:** 2011-12-13

**Authors:** Sabine Landau

**Affiliations:** 1 Kings College London, Institute of Psychiatry, Department of Biostatistics, London SE5 8AF, UK

## Background

Many mental health interventions constitute complex interventions since they are characterised by multiple components which when given in combination are thought to improve outcome as well as the components changing distal clinical outcome indirectly by inducing change in intermediate process variables (mediation). Ideally the evaluation of a complex intervention should be both pragmatic (assess effectiveness) as well as explanatory (measure the efficacy of the intervention components and separate their direct and indirect effects). Ordinary least squares (OLS) estimates of parameters of interest in explanatory analyses will suffer bias despite random treatment allocation when covariates are endogenous. In mental health trials such endogeneity is likely to arise as the result of non-compliance with randomised treatments, hidden confounding or measurement error in the treatment components received or process variables.

In this talk I discuss ways of designing a complex intervention trial so that all causal parameters of interest can be estimated consistently using instrumental variable (IV) methods.

## Methods

Pearl’s graphical checks of identifiability are applied to establish the most general causal model for mental health complex intervention trials under which parameters of interest remain identified.

Having determined this model, experimental actions including the generation of relevant IVs, that can ensure model assumptions hold, are reviewed.

Monte Carlo simulations are carried out to compare the performance of naïve OLS estimators with that of IV estimators.

## Results

Allowing for non-compliance with randomised treatment offers, hidden confounding or measurement errors requires independent randomisation of constituent treatment components and the availability of further instruments for hypothesised process variables. A number of avenues for generating the latter exist.

Simulations confirm that the biases suffered by OLS estimators are corrected by IV methods. The variance inflation of IV treatment component effect estimators is acceptable in standard sized mental health trials. However, instruments for process variables may be less strong raising the possibility that the variance inflation of mediation effect estimators renders them impractical for use.

## Conclusions

Investigators planning to carry out explanatory analyses of complex interventions should consider the generation of relevant IVs at the design stage.

The unbiased estimation of (total) effects of the treatment components actually received (efficacy) is feasible under non-compliance or treatment misclassification.

The generation of strong IVs for process variables may be more problematic. In mental health research mediation analyses allowing for hidden confounding and measurement error may only be feasible for large trials or after combining trials.

